# Pathway of care among psychiatric patients attending a mental health institution in central India

**DOI:** 10.4103/0019-5545.74308

**Published:** 2010

**Authors:** Chandrakant Lahariya, Shyam Singhal, Sumeet Gupta, Ashok Mishra

**Affiliations:** Department of Community Medicine, G.R. Medical College, and the associated J. A. Group of Hospitals, Gwalior, India; 1 Gwalior Mansik Arogyashala, Gwalior, India

**Keywords:** India, mental illnesses, pathway of care, psychiatry, social psychiatry

## Abstract

**Introduction:**

Only a limited proportion of patients with psychiatric disorders attend the healthcare facilities, and that too when the condition becomes severe. Treatment from unqualified medical practitioners and faith healers is a common practice, and is attributable to the delay in proper treatment.

**Materials and Methods:**

A cross-sectional study was conducted to understand the pathway of care adopted by psychiatric patients and its relationship with the socio-demographic determinants in the study population. The subjects were selected from urban specialty psychiatric hospitals and interviewed using a pre-tested, semi-structured interview schedule. The data was analyzed using SPSS v10.0 software. The Chi square test, T test, and Kruskall Wallis Test were used, as needed.

**Results:**

A total of 295 patients (203 males) were included in this study. The majority of the patients (45%) were suffering from Bipolar affective disorders (45%), followed by schizophrenia (36%). The majority, 203 (68%), were from the rural area, with 94 patients being illiterate. The mean distance traveled for treatment was 249 km. The majority of these (69%) had first contacted faith healers and a qualified psychiatrist was the first contacted person for only 9.2% of the patients.

**Conclusion:**

A large proportion of psychiatric patients do not attend any health facility due to a lack of awareness about treatment services, the distance, and due to the fear of the stigma associated with treatment. The psychiatric patients first seek the help of various sources prior to attending a psychiatric health facility. The pathway adopted by these patients need to be kept in mind at the time of preparation of the mental health program.

## INTRODUCTION

Mental illnesses are commonly linked with a higher disability and burden of disease, than many physical illnesses. The World Health Organization noted that one in every four people are affected by a mental disorder at some stage of life.[1] Six neuropsychiatry conditions, unipolar depressive disorders, alcohol use disorders, schizophrenia, bipolar affective disorder, Alzheimer’s, and other dementias, as also migraine, have figured in the top 20 causes of disability in the world.[2] It is estimated that at any point in time, in India, 2–5% of the population is suffering from serious mental illnesses, while another 10% of the population is suffering with minor mental illnesses.[3] In India, there are a very small number of qualified psychiatrists, mostly concentrated in the metropolitan and the urban areas, to deal with this huge problem, further compounding the issue.[4] Furthermore, it is a general observation in India that a majority of patients with mental disorder never seek professional help; and most of them utilize the help of unqualified medical practitioners, faith healers, and so on. The non-availability of mental health services, penury, stigma, and superstitions associated with mental disorders, coupled with the unwillingness or inability of families to care for their mentally ill relatives, appear to be the main contributory factors.[5] The widely prevalent magico-religious beliefs associated with mental illness and lower literacy, especially in rural areas, poses significant social obstacles in seeking appropriate health care for psychiatric patients.

The pathway a patient adopts to reach the appropriate treatment center is termed as the pathway of care. Studying the pathway of care helps us in analyzing health services use, identification of the sources of delay in attending the right care, and to find out the possible remedies. These care pathways are not random; they are structured by the convergence of psychosocial and cultural factors and have sufficient integrity to be studied directly as unfolding processes.[5]

In recent times, the pathway of care by psychiatric patients has been studied across the world.[6–9] However, there are limited studies from India, restricted to reputed hospitals and research centers.[10,11] Therefore, this study was planned, to understand the pathway of care adopted by psychiatric patients in central India. The present study was conducted to study the socio-demographic profile of psychiatric patients attending the Gwalior Mansik Arogyashala (GMA), a psychiatric specialty hospital in central India; to understand the pathways of care adopted by the patients attending this facility, and to explore the interrelationship between the pathways of care and the socio-demographic variables.

## MATERIALS AND METHODS

This is a hospital-based study, conducted at the Outpatient Department of a specialty psychiatric hospital (Gwalior Mansik Arogyashala) in Gwalior, India. This is a well-known, referral hospital in central India, and is affiliated to the medical college in the city. The Psychiatry Department of the medical college was located at this hospital, at the time of this study. The study subjects were the patients with psychiatric illnesses, who made their first contact with the study center, during the study period of May 2003 to April 2004.

Considering the time and resources available, it was decided that at least 250 patients would be included in this study. The data collection was based on the incidental sampling method,[12] where subjects were included for study by collecting the sample on stipulated days fixed beforehand. A pre-tested proforma along with the tool used in a WHO collaborative study, on the determinants of the outcome of severe mental disorders,[13] were used in this study. Although the WHO tool comprises of a series of boxes to mention the sequential contact held with different helping agencies in coded form, the second part of the pre-tested proforma was meant for collection of quantitative and qualitative information about the study subjects and the reasons for the delay in the care.

The two prefixed days of the week (Tuesday and Friday) were selected for data collection (this was decided as the researchers had the OPD on these days. However, this clinic was not for any specific psychiatric disorder and patients with any psychiatric disorder were seen). Only those subjects who had their first contact with the psychiatry outpatient clinic here were included in the study, after their informed consent. Those subjects who had been old cases at this facility or had attended any other specialty psychiatric health facility were not included, because such cases were not likely to have any recent contact with outside agencies and might have added to the recall bias. The data was collected by two (SS, SG) of the four authors of this study. The clinical information and the interview schedule were used for data collection by interviewing either the patient or the informant or both, as appropriate. (In case, it was noticed that patient was not able to give the correct history, the informants were interviewed and efforts were made to verify that information. The patients with psychotic disorder were less likely to give correct information, and therefore, information from the caregivers was also used). As the cases were worked up in detail; if at any stage of the study it was found that it was not the first contact with any psychiatric health facility, those subjects were not included in the final data analysis. The diagnosis was finally given, after complete history and examination, on the DSM-IV criteria, by one of the authors (SG), who is a qualified psychiatrist. The study protocol was approved by the institutional review board of the GMA and by the Medical College Hospital in Gwalior.

The data so collected was analyzed using the SPSS v10.0 software.[14] The basic characteristics of the subjects were presented as a proportion. For categorical variables, interdependence was tested by the chi square test. In some analysis, the groups were clubbed to meet the criteria for the chi square test. For scale variables, an independent sample t-test was used to determine the significance of the difference between the two means. For the grouping variable having more than two categories, the Kruskal Wallis test was used. A *P* value of <0.05 was considered statistically significant.

## RESULTS

A total of 304 subjects were enrolled in this study; however, the final analysis was done for 295 subjects (one patient refused to participate, and eight were excluded, as they were later on found to have attended another psychiatric health facility). Table 1 shows that the majority of these patients were in the age group of 16–45 years; two-thirds of them were male, another two-thirds were from the rural area, and one-third of the entire lot was illiterate. Most of these were suffering from severe mental illness; of which bipolar affective disorder 127 (45%) and schizophrenia 106 (35.9%) were the two most common ones.

**Table 1:** The first helping agency for psychiatric patients as per the socio-demographic characteristics (Qualitative)

Characteristics of patients	First helping agency					
	Psychiatrist	Allopathic practitioner	Traditional practitioner	Faith healer	Others	Total
	No. (%)	No. (%)	No. (%)	No. (%)	No. (%)	No.
Age group						
<15 Years	0	0	0	3(100)	0	3
16 – 45 Years	21 (8.1)	33 (12.8)	10 (3.9)	187 (72.5)	7 (2.7)	258
46 – 60 Years	6 (20)	14 (46.7)	2 (6.7)	8 (26.7)	0	30
> 60 Years	0	0	0	4 (100)	0	4
Sex						
Male	25 (12.3)	34 (16.7)	8 (3.9)	131 (64.5)	5 (2.5)	203
Female	2 (2.2)	13 (14.1)	4 (4.3)	71 (77.2)	2 (2.2)	92
Locality						
Rural	16 (7.9)	33 (16.3)	10 (4.9)	140 (69)	4 (2)	203
Urban	11 (12)	14 (15.2)	2 (2.2)	62 (67.4)	3 (3.3)	92
Diagnosis						
Acute psychosis	0	4 (18.2)	0	16 (72.7)	2 (9.1)	22
Schizophrenia	8 (7.5)	12 (11.3)	6 (5.7)	80 (75.5)	0	106
Bipolar affective disorder	10 (7.9)	17 (13.5)	6 (4.8)	88 (69.8)	5 (4)	126
Depression	6 (20.7)	8 (27.6)	0	15 (51.7)	0	29
Others	3 (25)	6 (50)	0	3 (25)	0	12
Total	27 (9.2)	47 (15.9)	12 (4.1)	202 (68.5)	7 (2.3)	295

Table 2 shows the mean year of education to be 6.25 years. The mean for years of education for male and female subjects was 7.3 years and 3.8 years, respectively (*P*=<0.05). The per capita monthly family income for the female subjects was lower than that for male subjects, with a statistical significant difference. (Females: mean income Rs. 334 / capita / month; Males: mean Rs. 473.0 / capita / month; *P*=<0.05). On the analysis of the distance traveled by these patients for treatment, it was noted that the mean distance traveled by these patients was 249 km.

**Table 2:** The first helping agency for psychiatric patients as per the socio-demographic characteristics (Quantitative)*

Characteristics (mean)	First helping agency						*P* value
	Psychiatrist	Allopathic practitioner	Traditional practitioner	Faith healer	Others	Total	
Distance from GMA (Kilometers)	333.12 (±39.8)	259.69 (±17.5)	417.25 (±106.1)	230.72 (±11.1)	98.57 (±52.1)	248.97 (±10.3)	=.001
Years of education	10.26 (±0.57)	8.71 (±0.8)	7.33 (±1.6)	5.16 (±0.32)	5.14 (±1.8)	6.25 (±0.29)	<.001
Per capita income (Rs.)	880.59 (±123.7)	411.1 (±33.1)	492.83 (±114.6)	372.33 (±17.5)	378.43 (±72.7)	429.81 (±19.7)	=.004
Delay at presentation (Months)	2.59 (±0.71)	4.53 (±1.1)	21.5 (±7.8)	12.62 (±1.3)	.29 (±0.18)	10.54 (±1.02)	58.01<.001
Total no. of patients	27 (9.2%)	47 (15.9%)	12 (4.1%)	202 (68.5%)	7 (2.4%)	295 (100%)	

* The values represent the mean values, while the figures in parenthesis depict standard deviations; GMA - Gwalior Mansik Arogyashala

Only 27 (9.2%) patients consulted a psychiatrist as the first helping agency, while 202 (68.5%) of the cases first consulted faith healers. Age group, gender, diagnosis, educational status, per capita income and distance from GMA, were the factors found to be statistically and significantly related to the first help sought [Tables 1 and 2]. The mean time patients took to reach the psychiatrist was found to be more when the first helping agencies were traditional healers (21.5 months) or faith healers (12.6 months), as compared to the other agencies. This difference was also significant statistically [Table 2].

**Figure 1:**
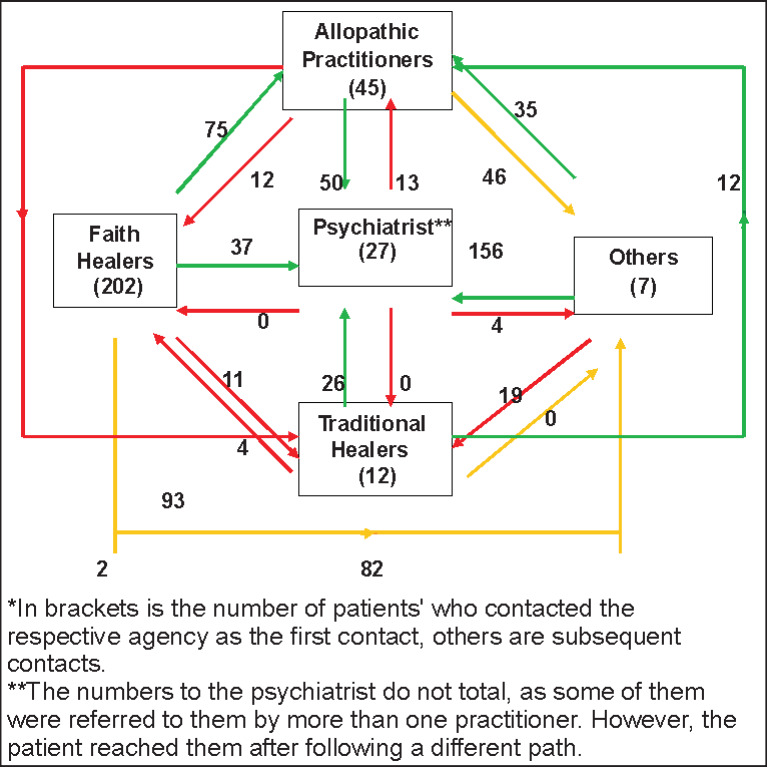
Pathways of care taken by psychiatric patients till they reached the psychiatric specialty hospital*

Figure 1 also shows that the faith healers were the most favorite agency for the first contact. While 202 patients contacted them as the primary helping agency, only 27 such contacts happened thereafter. Traditional healers, who were consulted by 12 patients initially, were later contacted by 35 more patients; 16 of these came to them after consulting faith healers, while another 19 from friends and other relatives. Other helping agencies (including previous patients) who were initially consulted by only seven patients, later emerged as the principal helping channels as 151 more cases took their help during subsequent contacts. Allopathic practitioners who were initially contacted by 47 patients, in the course of time had 102 more patients seeking their help. Most of these patients came to the allopathic practitioners after contacting faith healers. Psychiatrists were contacted as the first helping agency by only 27 patients. During subsequent contacts, the number of cases seeking the help of psychiatrists increased. It is evident from the diagram; major referring agency for the cases, for psychiatric help, was under the category ‘others,’ which includes previous patients (156 cases), followed by allopathic practitioners (87 cases).

The present study also found (data not in the tables) that 215 (73%) of these patients delayed health seeking due to the fear of stigma. One hundred and fifty-two (52%) were not aware of the place of appropriate treatment. Seventy-six (26%) thought that the symptom would resolve by itself. Fifty nine percent had shared their thoughts about the illness with their spouse. It was interesting to note that 35 (12%) of these patients had attended the clinic without the knowledge of family members.

Some qualitative information was also collected. A number of patients from rural areas reported the fear of stigmatization and isolation from the community if the psychiatric illness was disclosed, as the reason for the delay in treatment. The urban participants preferred to go to private practitioners as they believed that confidentiality was not maintained at government facilities. Some thought the treatment would be expensive and delayed the treatment.

## DISCUSSION

The possible reasons for most of the subjects in this study being 16 to 45 years may be because this is the economically productive age group; therefore, these patients have been brought for the right care. Nevertheless, the presentation of psychotic disorders more in the relatively younger age group (<40 years) has also been reported by authors in the past.[9–11]

This distribution in this study is highly skewed toward males (69%), which might be attributed to the prevailing gender bias in Indian society, where the illness of a male member is taken more seriously than that of a female patient. The findings in the present study also support the fact that psychiatric hospital services are utilized more by male patients than by female patients. Other studies from India have made similar observations.[15,16] The researchers have observed that these females are more likely to be illiterate, married, and from a lower income group[16,17] and the findings of our study corroborates the earlier reports.

The majority of patients in our study belonged to the lower socio-economic status, with low level of literacy, and was with a rural background. The study also noted that patients belonging to families from the urban, literate, and higher socio-economic status, preferred to take treatment from private practitioners or general hospital psychiatric setups. This could be due to the perceived stigma associated with mental illnesses and with that of psychiatric hospitals.

Predominance of psychotic illnesses (Schizophrenia, Bipolar affective disorder) among these patients may indicate that patients with minor and more common mental illnesses do not seek treatment from a specialty psychiatric hospital, as there is a common myth that psychiatric hospitals are for mentally ill persons, (*P*atients having psychotic disorders) rather than for patients with any other psychiatric illness. Similar findings have been reported by other authors in India,[18] and provide important lessons for the practitioners of psychiatric medicine in India.[19]

In the present study, for psychiatric illness, most cases contacted faith healers as the primary helping agency. However, subsequently, after not getting any relief, they sought the help of other agencies, such as, allopathic practitioners, traditional healers, and so on. The GMA was taken as a last resort when all other treatments had failed. A study on the treatment of psychiatric disorders in India observed that in view of the paucity of facilities, 80% of the population had to depend on indigenous treatments consisting of Ayurvedic and Unani systems of medicine, religious treatments consisting of prayers, fasting, and so on, as also various witchcrafts and magical rituals.[20] The situation is more or less the same even today, and not surprisingly 68.5% of the cases in our study contacted faith healers as the primary helping agency. Another study in India has noted that a substantial number of patients suffering from severe mental disorders seek non-professional care.[14] Although, the ancient wisdom may have some role in the treatment of mental disorders,[21] there is a need for generating awareness in the psychiatric patients in India to get professional help.

The traditional healers, while dealing with psychiatric patients, often hide their inability to understand and treat these disorders and attribute them to supernatural causes, further enhancing the misbeliefs of these patients. A study on the Indian indigenous healers[22] observed that relatively more healers than doctors revealed their diagnoses to the patient; and that the healers, when they did diagnose, did so in terms of ‘tick’, and ‘evil’ and treatment was largely with ashes, mullets, and holy water. Psychiatric patients used to go through different traditional and faith healers, including indigenous methods of exorcism, before arriving to proper care. This caused a delay in presentation, which was largely attributable to the stigma associated with such illnesses,[23] which in turn, led to suffering, and affected the outcome.

In our study, the delay in initiation of proper psychiatric treatment was noted. It was the long path that most of the patients took to reach GMA, and there was a significant delay of up to 10 years, with a mean delay of 10.5 months. Although it is an established fact that in a majority of psychiatric illnesses, (including schizophrenia, affective disorders) early diagnosis and treatment can significantly improve the outcome and prognosis, Gater *et al*[6] have noted that there is a longer delay on pathways involving native healers; while Gureje *et al*[7] and Banerjee and Banerjee from India,[24] have found that the patients who first consulted traditional healers, tended to arrive at a tertiary psychiatric service much later than those who consulted other caregivers. The results of our study corroborate these findings.

Another important point noted in our study was that the previous patients and their relatives (included in category ‘others’) were the major referring agency for patients to go for psychiatric care followed by allopathic practitioners. A study in 1998,[25] observed that 25% of the patients took their own initiative to seek help and more than 55.2% were referred by the spouse or relatives; while another study[26] observed that the medical sector was the most common source of referral. The present study was conducted at a referral facility, therefore, these was a possibility in this study that a majority of the patients referred from a great distance, were found to have a severe form of psychiatric illness.

The limitation of this study was that it was conducted at a mental hospital only; a study from a medical college hospital, where more representative population attends the OPDs or a multicentric study could have given different results. Secondly, prefixed days are usually attended by the same specialist always; therefore, attendance to such prefixed days may also depend upon the perceived expertise and professional reputation of the expert, among the general population. Third, the patient groups used in this study were heterogenous and the pathway of care may have affected them, according to the disorder.

## CONCLUSION

The study found that the majority of patients attending the mental hospital suffered from severe mental illnesses and belonged to the male gender, rural locality, lower socio-economic class, and was with a low educational status. Faith healers were the most commonly sought primary helping agency among the study subjects. Pathways involving faith healers and traditional healers took a longer time to reach the right psychiatric help. The need for incorporating an efficient and effective referral mechanism, the role of various service providers in the pathway of care, and availability of services should kept in mind when preparing any mental health program in India.

## ANNEXURE

### Definitions

1. The Psychiatrist in this study was the person who had completed three years of postgraduation studies in Psychiatric medicine from an institute recognized by the Medical Council of India.
2. Allopathic Practitioner was anybody who had at least completed his graduation in Medicine and received the degree of MBBS from any medical college in India. However, this person would not have specialized in Psychiatric Medicine.
3. Traditional practitioner was anybody who had qualified in any stream of medicine except Allopathic medicine. Government of India recognizes Ayurveda, Unani, Siddha, and Homeopathy as standard streams of medicine. The practitioners of any of these streams were considered as traditional practitioners in this study.
4. Faith healers were the people who practiced witchcraft, and treated patients by using magico-religious practices. They did not have any medical qualification.
5. Others were the relatives / friends of the study subjects. The previous patients with such illnesses were also included in this group, in the present study.
